# Association of Tau and Amyloid with Representational Similarity in the Amygdala’s Reactivity to Negative and Neutral Stimuli in Individuals at Risk for Alzheimer’s Disease

**DOI:** 10.1002/alz.094956

**Published:** 2025-01-09

**Authors:** Mingtong Liu, Tobey J. Betthauser, Lauren K. Gresham, Stacey M. Schaefer

**Affiliations:** ^1^ Institute on Aging, University of Wisconsin‐Madison, Madison, WI USA; ^2^ Department of Medicine, University of Wisconsin‐Madison School of Medicine and Public Health, Madison, WI USA; ^3^ Wisconsin Alzheimer’s Disease Research Center, School of Medicine and Public Health, University of Wisconsin‐Madison, Madison, WI USA

## Abstract

**Background:**

Emotional dysfunction is often observed in older adults at the early stage of Alzheimer’s disease. Individuals at risk for Alzheimer’s disease may show differences in emotional reactivity before they exhibit cognitive decline (Fredericks et al., 2018). It is unclear whether emotional symptoms are associated with brain changes during the development of the disease. In this study, we examined the associations of tau and amyloid burden with representational similarity in the amygdala’s reactivity to negative and neutral stimuli in individuals at risk for Alzheimer’s disease.

**Method:**

Participants are from the Wisconsin Registry for Alzheimer Prevention (n = 81, 69% Female, 6% BIPOC). During the fMRI scans, participants viewed 30 negative, 30 neutral, and 30 positive images followed by a neutral face. Representational similarity analysis was used to calculate the similarity of the amygdala activation pattern to negative images and neutral images. Amyloid and tau burden were measured using the positron emission tomography.

**Result:**

Our results showed that greater tau burden in the entorhinal cortex is significantly related to less neural similarity in right amygdala to negative and neutral stimuli. Individual differences of tau burden in the entorhinal cortex, but not amyloid burden, significantly predict neural similarity in the right amygdala to negative and neutral stimuli when controlling for covariates, such as age, gender, and race. Neural similarity in both left and right amygdala to negative and neutral stimuli is greater in the T− group, compared to the T+ group.

**Conclusion:**

Greater tau burden in the entorhinal cortex predicts less neural similarity in the right amygdala to negative and neutral stimuli, suggesting a greater differentiation of the amygdala’s response with higher tau.

